# Tigecycline Heteroresistance and Resistance Mechanism in Clinical Isolates of Acinetobacter baumannii

**DOI:** 10.1128/Spectrum.01010-21

**Published:** 2021-09-15

**Authors:** Jeongwoo Jo, Kwan Soo Ko

**Affiliations:** a Department of Microbiology, Sungkyunkwan University School of Medicine, Suwon, Republic of Korea; University of Texas Southwestern Medical Center

**Keywords:** *Acinetobacter baumannii*, tigecycline, heteroresistance, efflux pump

## Abstract

Tigecycline is regarded as a last-resort treatment for multidrug-resistant Acinetobacter baumannii. However, tigecycline resistance in A. baumannii has increased worldwide. In this study, we investigated tigecycline heteroresistance in A. baumannii isolates from South Korea. Antibiotic susceptibility testing was performed on 323 nonduplicated A. baumannii isolates. Among 260 and 37 tigecycline-susceptible and -intermediate-resistant A. baumannii isolates, 146 (56.2%) and 22 (59.5%) isolates were identified as heteroresistant to tigecycline through a disk diffusion assay and population analysis profiling. For selected isolates, an *in vitro* time-kill assay was performed, and survival rates were measured after preincubation with diverse concentrations of tigecycline. Heteroresistant isolates showed regrowth after 12 h of 2× MIC of tigecycline treatment, and resistant subpopulations were selected by preexposure to tigecycline. Furthermore, genetic alterations in *adeABC, adeRS*, and *rpsJ* were assessed, and the relative mRNA expression levels of *adeB* and *adeS* were compared. The tigecycline resistance in subpopulations might be due to the insertion of IS*Aba1* in *adeS*, leading to the overexpression of the AdeABC efflux pump. However, the tigecycline resistance of subpopulations was not stable during serial passages in antibiotic-free medium. The reversion of tigecycline susceptibility by antibiotic-free passages might occur by additional insertions of IS*Aba10* in *adeR* and nucleotide alterations in *adeS* in some mutants. Tigecycline heteroresistance is prevalent in A. baumannii isolates, which results in treatment failure. Tigecycline resistance is mainly due to the overexpression of the AdeABC efflux pump, which is associated with genetic mutations, but this resistance could be reversed into susceptibility by additional mutations in antibiotic-free environments.

**IMPORTANCE** The evidence that antibiotic heteroresistance is responsible for treatment failure in clinical settings is increasing. Thus, detection and characterization of heteroresistance would be important for appropriate therapeutic guidance to treat bacterial infections. However, data on tigecycline heteroresistance in Gram-negative bacteria is currently limited, although tigecycline is regarded as a last-line antibiotic against infections caused by antibiotic-resistant pathogens. In this study, we investigated the tigecycline heteroresistance in Acinetobacter baumannii, which has been listed by the WHO as a priority for research and development of new antibiotics. We found very high prevalence of tigecycline-heteroresistant A. baumannii clinical isolates, which may result in treatment failure due to the selection of resistant subpopulations. We also identified the main resistance mechanism in tigecycline-resistant subpopulations, that is, upregulation of AdeABC efflux pumps due to IS*Aba1* insertion in *adeS.*

## INTRODUCTION

Acinetobacter baumannii is a major nosocomial pathogen responsible for a range of infectious diseases, such as bacteremia and pneumonia (1). In addition, it is a member of the ESKAPE (Enterococcus faecium, Staphylococcus aureus, Klebsiella pneumoniae, Acinetobacter baumannii, Pseudomonas aeruginosa, and Enterobacter species) group, which is becoming increasingly resistant to commonly used antibiotics (2). Because of the intrinsic resistance to numerous antibiotics and the ability to readily acquire new resistance determinants, a high percentage of A. baumannii isolates are resistant to a wide range of antibiotics (1). Due to the resistance to multiple antibiotics, including carbapenems, A. baumannii has been classified by the World Health Organization as a priority on the global priority list for research and development of new antibiotics (3). There are only a few therapeutic options for treating infections caused by multidrug-resistant (MDR) A. baumannii, including polymyxins and tigecycline. However, in addition to polymyxin-resistant isolates, tigecycline resistance has been increasingly reported in A. baumannii (4).

Tigecycline was the first member of the glycylcycline class and was derived from tetracycline; tigecycline was designed to overcome the common mechanism of tetracycline resistance (5). It inhibits bacterial growth by binding to the bacterial 30S ribosome and by blocking the entry of tRNA, eventually preventing protein synthesis. In addition, tigecycline evades tetracycline resistance mechanisms because its binding orientation is different from that of tetracyclines (6). Although tigecycline is regarded as a last-line antibiotic against infections caused by MDR or extensively drug-resistant (XDR) bacterial pathogens, tigecycline resistance has been reported worldwide (7). A. baumannii acquires tigecycline resistance by overexpression of efflux pumps, especially AdeABC, and modification of tigecycline-binding sites in ribosomes by *rpsJ* mutation (8).

Antibiotic heteroresistance is defined as a mixed population of antibiotic-susceptible and antibiotic-resistant bacteria in a single bacterial isolate (9). Subpopulations of susceptible isolates exhibit reduced susceptibility to the corresponding antibiotics. Although the clinical relevance of antibiotic heteroresistance is unclear, there is increasing evidence that heteroresistance is responsible for treatment failure due to the selection of resistant subpopulations after antibiotic treatment (10). Thus, the detection and characterization of heteroresistance can provide appropriate therapeutic guidance for antibiotic treatment. Tigecycline heteroresistance has been investigated in only a few Gram-negative bacterial species, including Salmonella enterica serovar Typhimurium and Enterobacter cloacae (11, 12), but it has not been investigated in A. baumannii.

In this study, we investigated the proportion of tigecycline heteroresistance among clinical isolates of A. baumannii from South Korea. In addition, we characterized the tigecycline-heteroresistant A. baumannii isolates and their respective resistant subpopulations and explored the tigecycline resistance mechanism in resistant subpopulations.

## RESULTS

### Identification of tigecycline heteroresistance.

**FIG 1:**
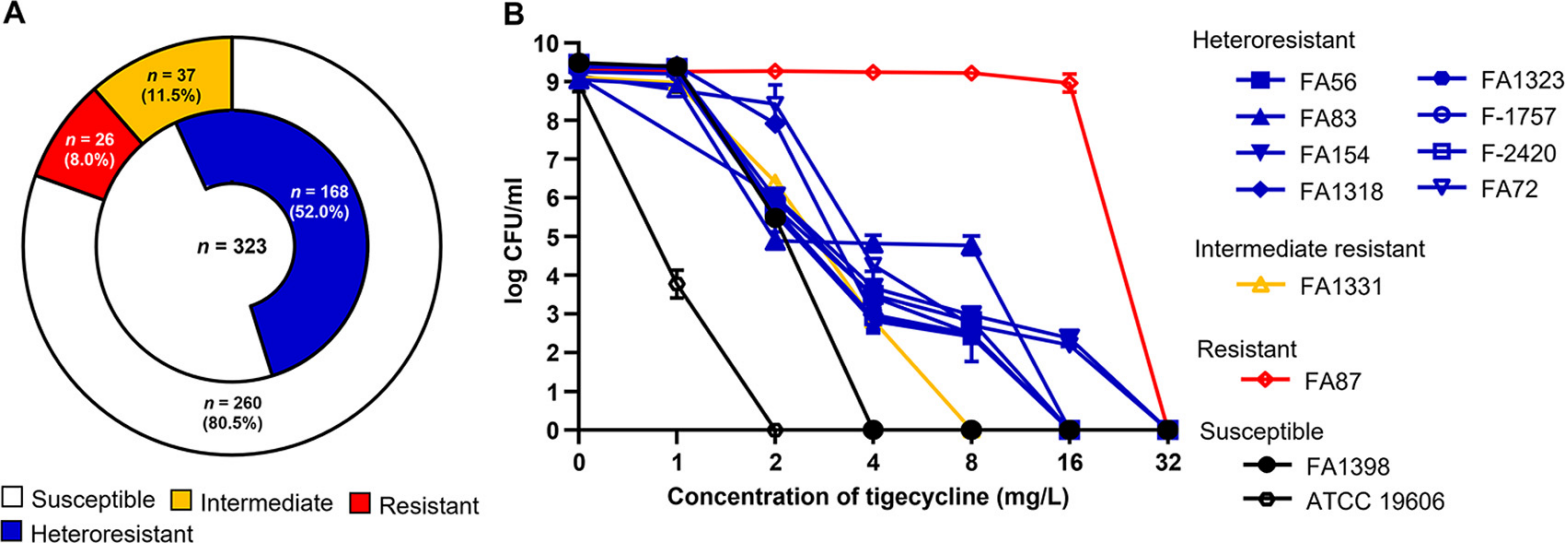
(A) Tigecycline susceptibilities of A. baumannii isolates in this study and ratios of tigecycline heteroresistance in tigecycline-susceptible and -intermediate-resistant isolates. (B) Results of population analysis profiling (PAP) in selected A. baumannii isolates; eight heteroresistant (seven from susceptible and one from an intermediate-resistant isolate) and four homogenous (two susceptible, one intermediate-resistant, and one resistant) isolates were included.

As a result of tigecycline susceptibility testing, 26 A. baumannii clinical isolates (8.0%) were found to be resistant to tigecycline (Fig. 1A). Thirty-seven isolates (11.5%) were intermediate resistant to tigecycline, and 260 isolates (80.5%) were susceptible to it. Among the 260 tigecycline-susceptible isolates, 146 (56.2%) were determined to be heteroresistant based on population analysis profiling (PAP). Twenty-two of 37 tigecycline intermediate-resistant isolates (59.5%) were also identified as heteroresistant to tigecycline. Overall, 168 isolates were heteroresistant to tigecycline (Fig. 1A).

Table 1 and Fig. 1B display the results of tigecycline susceptibility testing and PAP assay of randomly selected isolates, which were used for further studies. The resistant subpopulations of the heteroresistant isolates that were obtained during the PAP assay exhibited 8-fold-higher MICs than the original isolates (Table 1).

**TABLE 1:** Genotypes and MICs of tigecycline against parental strains and their resistant subpopulations in the absence or presence of efflux pump inhibitors

Population-level resistance, isolate	Specimen type	ST^*a*^	TIG MIC (mg/liter)^*b*^			
			P	RP	RP + CCCP	RP + PAβN
Heteroresistant						
FA56	Sputum	191	2	16	4	8
FA83	Sputum	191	2	16	0.5	8
FA154	Sputum	191	2	16	0.5	8
FA1318	Sputum	357	2	16	4	8
FA1323	Sputum	357	2	16	2	8
F-1757	Blood	357	2	16	2	8
F-2420	Blood	357	2	16	1	8
FA72	Sputum	191	4	32	2	8

Homogeneous						
FA1398	Sputum		2			
FA1331	Sputum		4			
FA87	Sputum		64			
ATCC 19606			1			

a ST, sequence type.

b TIG, tigecycline; P, parental strain; RP, resistant population.

### Efficacy of tigecycline against heteroresistant isolates.

**FIG 2:**
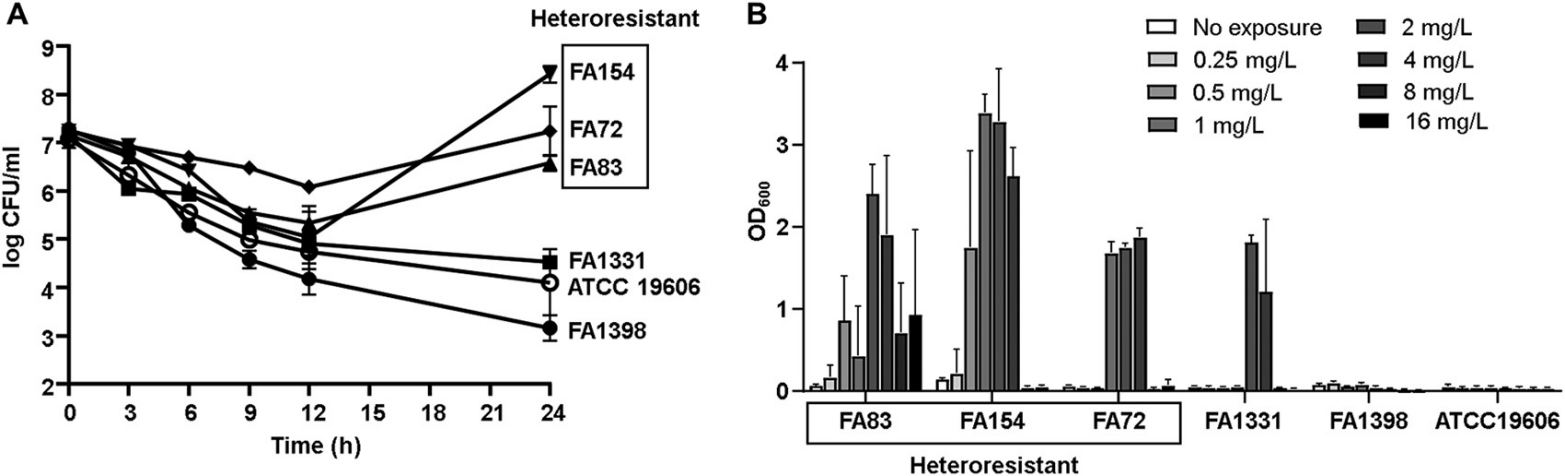
(A) Results of the *in vitro* time-kill assay. Three heteroresistant (two from susceptible and one from an intermediate-resistant isolate) and three homogenous (two susceptible and one intermediate-resistant) isolates were included in the assay. (B) Selection of resistant populations by preexposure to diverse concentrations of tigecycline. The survival rates were measured by their absorbance at 600 nm (OD, optical density).

We performed an *in vitro* time-kill assay to evaluate the antibiotic efficacy of tigecycline in heteroresistant isolates belonging to two different clones, sequence type 191 (ST191) and ST357. To compare efficacies, we included homogeneously susceptible and intermediate-resistant isolates in the assay. The three tigecycline-heteroresistant A. baumannii isolates were killed by 2× MIC of tigecycline more slowly than the homogeneously susceptible isolates and showed regrowth after 12 h of tigecycline treatment (Fig. 2A). In contrast, no regrowth was observed in homogeneously susceptible and intermediate-resistant isolates.

The effect of previous tigecycline exposure on the selection of resistant populations was investigated (Fig. 2B). In tigecycline-heteroresistant isolates, tigecycline preexposure was shown to select tigecycline-resistant populations. The selection of resistant populations was maximal on exposure to moderate concentrations of tigecycline (1 to 4 mg/liter). In the homogeneously susceptible isolates, resistant populations were not selected by preexposure to antibiotics. However, resistant populations were identified by exposure to 2 and 4 mg/liter tigecycline in a tigecycline-intermediate-resistant isolate, FA1331.

### Stability of tigecycline resistance of resistant subpopulations.

We investigated the stability of tigecycline resistance in resistant subpopulations obtained from tigecycline-heteroresistant isolates in antibiotic-free medium (Table 2). After 30 days of passaging, the tigecycline MICs of all resistant subpopulations (RP) decreased gradually, although the rates of MIC decrease differed. However, three colonies recovered after 30 days of passage (RP-p30 mutants) showed tigecycline intermediate resistance, and FA83-RP-p30 preserved its tigecycline MIC over the breakpoint of resistance.

**TABLE 2:** Changes of tigecycline MICs in tigecycline-resistant populations of heteroresistant isolates after serial passaging in antibiotic-free medium

Isolate	TIG MIC (mg/liter) after indicated duration of passage (days)^*a*^						
	0	5	10	15	20	25	30
FA56-RP	16	8	4	4	4	4	4
FA83-RP	16	8	8	8	8	8	8
FA154-RP	16	16	16	8	4	4	2
FA1318-RP	16	16	16	16	16	2	2
FA1323-RP	16	8	8	8	8	8	4
F-1757-RP	16	8	8	8	8	8	2
F-2420-RP	16	8	8	8	8	4	2
FA72-RP	32	16	16	8	4	4	4

a White number on black background, resistant; gray background, intermediate resistant; white background, susceptible.

### Effect of efflux pumps in resistant subpopulations.

To investigate the effect of efflux pumps on tigecycline resistance in resistant subpopulations, efflux pump inhibitors were treated and tigecycline MICs were measured. Two efflux pump inhibitors, CCCP (carbonyl cyanide 3-chlorophenylhydrazone) and PAβN (phenyl-arginine-β-naphthylamide dihydrochloride), showed somewhat different results: 16 mg/liter of CCCP reduced tigecycline MICs by 4-fold to 32-fold, but the tigecycline MICs were reduced 2-fold by 50 mg/liter of PAβN (Table 1). In FA87, a homogeneously resistant isolate (MIC of 64 mg/liter), the MICs were reduced 16 mg/liter and 32 mg/liter by CCCP and PAβN, respectively.

**FIG 3:**
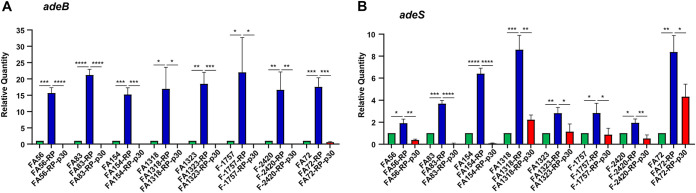
Relative mRNA expression levels of *adeB* and *adeS* in the wild-type isolates (FA#), tigecycline-resistant subpopulations (FA#-R), and mutants obtained after serial passaging in antibiotic-free medium (FA#-R-p30). Expression was measured as a relative quantity by qRT-PCR using the *rpoB* gene as the reference.

The relative expression levels of *adeB* and *adeS*, which are well-known efflux pump genes associated with tigecycline resistance in A. baumannii, were compared among the original isolates, resistant subpopulations, and colonies obtained from resistant subpopulations after 10, 20, and 30 days of passage in antibiotic-free medium (Fig. 3 and Fig. S1 in the supplemental material). The resistant subpopulations showed significantly increased expression of both genes. The expression levels of the genes gradually decreased during passages in antibiotic-free medium.

Genetic alterations of *adeABC* and *adeRS* were investigated in resistant subpopulations and RP-p10, PR-p20, and RP-p30 mutants. In all resistant subpopulations, no mutations were identified in *adeR*, but IS*Aba1* was detected in *adeS* (Table 3). IS*Aba1* insertions in resistant subpopulations were preserved in the RP-p10, RP-p20, and RP-p30 mutants. Additional mutations were identified during the serial passages in antibiotic-free passages. IS*Aba10* insertions were identified in RP-p30 mutants from FA56 in *adeR*, and they were found in RP-p20 and RP-p30 mutants from FA83 and FA154. An insertion of a nucleotide was identified in *adeS* of F-2420-RP-p30. No mutations in *adeABC* and *rpsJ* were identified in either resistant or RP-p30 mutants. *tet*(X) orthologues were not detected in our resistant populations.

**TABLE 3:** Genetic alterations of efflux pump regulatory genes *adeR* and *adeS* detected in resistant subpopulations and in colonies recovered after different durations of serial passaging compared with sequences of parental strains

Isolate	TIG MIC^*a*^ (mg/liter)		Genetic alteration(s) in indicated subpopulation^*b*^							
			*adeR*				*adeS*			
	RP	RP-p30	RP	RP-p10	RP-p20	RP-p30	RP	RP-p10	RP-p20	RP-p30
FA56	16	4	ND	ND	ND	IS*Aba10* (nt 402)	IS*Aba1* (nt 371)	IS*Aba1* (nt 371)	IS*Aba1* (nt 371)	IS*Aba1* (nt 371)
FA83	16	8	ND	ND	IS*Aba10* (nt 200)	IS*Aba10* (nt 200)	IS*Aba1* (nt 379)	IS*Aba1* (nt 379)	IS*Aba1* (nt 379)	IS*Aba1* (nt 379)
FA154	16	2	ND	ND	IS*Aba10* (nt 52)	IS*Aba10* (nt 52)	IS*Aba1* (nt 371)	IS*Aba1* (nt 371)	IS*Aba1* (nt 371)	IS*Aba1* (nt 371)
FA1318	16	2	ND	ND	ND	ND	IS*Aba1* (nt 371)	IS*Aba1* (nt 371)	IS*Aba1* (nt 371)	IS*Aba1* (nt 371)
FA1323	16	4	ND	ND	ND	ND	IS*Aba1* (nt 430)	IS*Aba1* (nt 430)	IS*Aba1* (nt 430)	IS*Aba1* (nt 430)
F-1757	16	2	ND	ND	ND	ND	IS*Aba1* (nt 379)	IS*Aba1* (nt 379)	IS*Aba1* (nt 379)	IS*Aba1* (nt 379)
F-2420	16	2	ND	ND	ND	ND	IS*Aba1* (nt 422)	IS*Aba1* (nt 422)	IS*Aba1* (nt 422)	IS*Aba1* (nt 422), 2195_2196insT
FA72	32	4	ND	ND	ND	ND	IS*Aba1* (nt 371)	IS*Aba1* (nt 371)	IS*Aba1* (nt 371)	IS*Aba1* (nt 371)

a TIG, tigecycline; RP, resistant population; RP-p30, colonies recovered from tigecycline-resistant population after serial passaging of 30 days in antibiotic-free LB broth.

b RP-p10 and RP-p20, colonies recovered from tigecycline-resistant population after serial passaging of 10 and 20 days, respectively, in antibiotic-free LB broth; ND, not detected; nt, nucleotide; 2195_2196insT, insertion of T between nucleotides 2195 and 2196.

## DISCUSSION

Antibiotic heteroresistance describes a heterogeneous strain with subpopulations exhibiting increased levels of antibiotic resistance compared to that of the main population (9). Although it is somewhat disputable, it is well-known that heteroresistance is one of the causes of treatment failure. Thus, the detection and characterization of heteroresistance in clinical isolates is critical for maximizing the efficacy of antibiotics (10). However, there have been only a few reports on heteroresistance to tigecycline. In the present study, we investigated tigecycline heteroresistance in clinical A. baumannii isolates belonging to the ESKAPE group (2).

In this study, more than half of the clinical A. baumannii isolates that were determined to be susceptible or intermediate resistant to tigecycline were identified as tigecycline heteroresistant. We identified heterogeneous populations of intermediate-resistant isolates. This indicates that tigecycline heteroresistance might be prevalent among A. baumannii isolates.

It was demonstrated that the antibiotic efficacy of tigecycline would be reduced in tigecycline-heteroresistant A. baumannii isolates. In the *in vitro* time-kill assay, all tigecycline-heteroresistant isolates were not removed by 2× MIC of tigecycline, and they showed regrowth after 12 h of antibiotic treatment. Consistent with the results of the *in vitro* time-kill assay, exposure to various concentrations of tigecycline resulted in increases in tigecycline-resistant populations. The increases in resistant populations were obvious after exposure to 1 to 4 mg/liter tigecycline, which corresponds to the range of tigecycline concentrations administered to patients (13, 14). The selection of resistant populations in an intermediate-resistant isolate, FA1331, was unexpected. In FA1331, populations that survived in 8 mg/liter of tigecycline were observed after exposure to 2 and 4 mg/liter of tigecycline. Although PAP could not identify heteroresistance in the isolate, resistant populations might exist at a very low frequency in the original isolate. Otherwise, the concentration within the mutation selection window might lead to tigecycline resistance (15, 16). Coupled with the finding that tigecycline heteroresistance may be prevalent in A. baumannii isolates, the low efficacy of tigecycline in *in vitro* time-kill assays suggests that it is necessary to develop a fast and accurate method to detect heteroresistance.

Many studies have suggested that resistance-nodulation-division (RND) efflux pumps, including AdeABC, are associated with tigecycline resistance (8, 17). We confirmed the role of RND efflux pumps in tigecycline-resistant populations of heteroresistant A. baumannii isolates. In all resistant populations selected in this study, the insertion of IS*Aba1* in the *adeS* gene was identified at different sites, and the mRNA expression of *adeB* and *adeS* was upregulated. IS*Aba1* insertion in the *adeS* gene generates a truncated soluble AdeS protein by the *P*_out_ promoter on IS*Aba1*, which contributes to the overexpression of the AdeABC pump, leading to tigecycline resistance (18).

Tigecycline resistance in resistant populations was not stable. Although the degrees and speeds of MIC reduction were different, tigecycline MICs decreased with passage in antibiotic-free medium. The mRNA expression levels of *adeB* and *adeS* also decreased, confirming that tigecycline resistance in resistant populations was due to the function of the AdeABC efflux pump. In some mutants, the restored susceptibility to tigecycline might be due to additional mutations, such as IS*Aba10* insertion in *adeR* or insertion of a nucleotide in *adeS*. However, no additional mutations in *adeABC* and *adeR* were identified in the other mutants. Mutations in other genes may be associated with restored susceptibility, or phenotypic changes without genetic alterations might occur during the passages. Further studies on the mechanisms of reversion to tigecycline susceptibility would lead to findings of other tigecycline resistance mechanisms. Furthermore, it could lead to finding a way to prevent the emergence of tigecycline resistance.

In short, we found that tigecycline heteroresistance was present in a very high proportion of A. baumannii clinical isolates. It was revealed that tigecycline heteroresistance would result in treatment failure due to the selection of resistant populations by exposure to a moderate concentration of tigecycline. The upregulation of AdeABC efflux pumps due to IS*Aba1* insertion in *adeS* might be the main resistance mechanism in resistant populations, but the resistance decreased during serial passages in antibiotic-free medium due to additional mutations in *adeR* and *adeS* or to unknown reasons.

## MATERIALS AND METHODS

### Bacterial isolates and tigecycline susceptibility testing.

A total of 323 nonduplicated A. baumannii isolates were collected between 2011 and 2015 from patients at Daegu Fatima Hospital (Daegu, South Korea). Among them, 252 isolates (78.0%) were from sputum samples, followed by blood samples (38 isolates, 11.8%). Others were from wound, urine, bronchial aspirate, and ear samples. Approval by Institutional Review Board or Institutional Animal Care and Use Committee was not required because our research was not involved with human subjects or animal experimentation.

MICs of tigecycline were determined for all A. baumannii isolates by the standard broth microdilution method according to the guidelines of the Clinical and Laboratory Standards Institute (CLSI) (19). Since there are no established MIC breakpoints for tigecycline in Acinetobacter spp., each isolate was classified as tigecycline susceptible, intermediate, or resistant based on the latest FDA-identified interpretive criteria for *Enterobacteriaceae*: a MIC of ≤2 mg/liter was classified as susceptible, a MIC of 4 mg/liter was classified as intermediate, and a MIC of ≥8 mg/liter was classified as resistant (20). Escherichia coli ATCC 25922 was used as the reference strain. For selected tigecycline-heteroresistant isolates, the MICs of their respective tigecycline-resistant subpopulations were also determined by the same method.

### Determination of tigecycline heteroresistance.

For tigecycline-susceptible and -intermediate A. baumannii isolates, tigecycline heteroresistance was determined by two independent methods: disk diffusion assay and population analysis profiling (PAP). The disk diffusion assay was performed according to the CLSI recommendations (21). A 15-μg tigecycline disk (Becton, Dickinson and Company, Franklin Lakes, NJ, USA) was placed on the surface of a cation-adjusted Mueller-Hinton (MH II) agar plate inoculated with 0.5 McFarland standard bacterial suspension. After 24 h of incubation at 37°C, colony growth within the inhibitory zone was deemed to be tigecycline heteroresistance. For PAP, aliquots of 20 μl of 10-fold serially diluted overnight cultures of all isolates shaken at 185 rpm at 37°C were spread on MH II agar plates with tigecycline concentrations in a 2-fold-change gradient from 1 to 32 mg/liter. After 24 h of incubation at 37°C, the CFU/ml was calculated. Tigecycline heteroresistance was defined as a population containing resistant subpopulations with MICs at least 8-fold higher than their respective parental strains at frequencies of 10^−6^ to 10^−7^ (9). Tigecycline-resistant subpopulations were obtained from colonies grown on agar plates with the highest concentrations of tigecycline in the PAP assay and stored as frozen stock. Tigecycline-susceptible A. baumannii ATCC 19606 was used as a control.

### *In vitro* time-kill assay and multilocus sequence typing (MLST).

The antibiotic efficacy of tigecycline against tigecycline-heteroresistant isolates was evaluated by an *in vitro* time-kill assay and compared with the efficacy against homogeneously tigecycline-susceptible and -intermediate isolates according to the CLSI guidelines (22). Briefly, overnight cultures of each of the three selected tigecycline-heteroresistant, -susceptible, and -intermediate-resistant isolates were diluted 1:100 and exposed to tigecycline at 2-fold the MICs in MH II broth. Viable cells were determined at 0, 3, 6, 9, 12, and 24 h by a spotting test (23). A. baumannii ATCC 19606 was used as a reference strain.

For the tigecycline-heteroresistant A. baumannii isolates analyzed in the *in vitro* time-kill assay, we performed MLST as described at https://pubmlst.org/bigsdb?db=pubmlst_abaumannii_oxford_seqdef (24).

### Measurement of survival rate after preincubation with tigecycline.

The survival rate of the tigecycline-resistant subpopulation was measured after exposure or nonexposure to subinhibitory concentrations of tigecycline according to the method of a previous study (25), with some modifications. Overnight cultures of tigecycline-heteroresistant, -susceptible, and -intermediate, A. baumannii isolates grown in MH II broth were inoculated at a 1:100 ratio into fresh medium and treated with tigecycline at 0, 0.25, 0.5, 1, 2, 4, 8, and 16 mg/liter. After incubation for 24 h at 37°C with shaking at 185 rpm, each bacterial culture was inoculated onto MH II broth with 8 mg/liter of tigecycline to identify the influence of treatments with diverse concentrations on the selection of resistant subpopulations among the whole population. After 24 h of incubation at 37°C and 185 rpm, the survivability was evaluated by measuring the absorbance at 600 nm using a GeneQuant 1300 spectrophotometer (Biochrom, Cambridge, UK).

### Stability test for tigecycline resistance.

To investigate the stability of tigecycline MICs of tigecycline-resistant subpopulations obtained from each tigecycline-heteroresistant A. baumannii isolate, the surviving colonies on MH II agar containing 8 mg/liter of tigecycline were incubated in Luria-Bertani (LB) broth without antibiotics at 37°C at 185 rpm and diluted 1:100 in fresh LB broth every 24 h for 30 days. The stability of tigecycline-resistant subpopulations was estimated by comparing tigecycline MICs.

### Efflux pump inhibitor assay.

To investigate the effect of efflux pumps on tigecycline resistance, changes in MICs were monitored in the presence of efflux pump inhibitors (16 mg/liter of CCCP [carbonyl cyanide 3-chlorophenylhydrazone; Sigma-Aldrich, St. Louis, MO, USA] or 50 mg/liter of PAβN [phenyl-arginine-β-naphthylamide dihydrochloride; Sigma-Aldrich, St. Louis, MO, USA]). MICs were measured by broth microdilution as previously described.

### Sequencing for *adeABC*, *adeRS*, and *rpsJ*.

The genetic alterations in the efflux pump gene *adeABC* and its transcriptional regulatory gene, *adeRS*, as well as *rpsJ*, coding for 30S ribosomal protein S10, and *tet*(X), were assessed by nucleotide sequencing. Genomic DNA was extracted from the wild-type isolates (FA#), tigecycline-resistant subpopulations (FA#-R), and mutants obtained after serial passaging in antibiotic-free medium (FA#-R-p30) using the G-spin genomic DNA extraction kit for bacteria (iNtRON Biotechnology, Seongnam, Korea). PCR and DNA sequencing using the Sanger method were performed using the primers listed in Table S1. The genetic alterations were analyzed using SnapGene version 4.1.9 (GSL Biotech LLC, Chicago, IL, USA).

### qRT-PCR.

The relative mRNA expression levels of *adeB* and *adeS* in the wild-type isolates (FA#), tigecycline-resistant subpopulations (FA#-R), and mutants obtained after serial passaging in antibiotic-free medium (FA#-R-p#) were compared by qRT-PCR. All strains analyzed were grown in LB broth until mid-log phase. Total RNA was extracted using the RNeasy minikit (Qiagen, Hilden, Germany) and converted to cDNA using the HiSenScript RH[-] RT premix kit (iNtRON Biotechnology, Seongnam, Korea). Quantitative reverse transcription-PCR (qRT-PCR) was performed in triplicate using TB Green premix Ex Taq (TaKaRa, Kyoto, Japan) and the QuantStudio 7 flex real-time PCR system (Thermo Fisher Scientific, Waltham, MA, USA). The mRNA expression levels of target genes were measured relative to that of the reference gene *rpoB* using the cycle threshold (ΔΔ*C_T_*) method.

### Statistical analysis.

Statistical analyses were conducted by Student’s *t* test using Prism version 8.3.0 software for Windows (GraphPad Software). Statistical difference was considered significant at a *P* value of <0.05 (*, *P < *0.05; **, *P < *0.001; ***, *P < *0.0001).
