# Supplementary material for: Tigecycline Heteroresistance and Resistance Mechanism in Clinical Isolates of Acinetobacter baumannii
Source: Microbiol Spectr. 2021 Sep 15;9(2):e01010-21. doi: 10.1128/Spectrum.01010-21 (PMC8557860; doi:10.1128/Spectrum.01010-21)

**Supplementary Table S1.** Primers used in this study

| Primers | Sequence (5′-3′) |
|---|---|
| Primers for sequencing | |
| *adeA*-F | ATGCAAAAGCATCTTTTACTTCCT |
| *adeA*-R | TCATGGTTGTGCCCCTTCAG |
| *adeB*-F | ATGATGTCACAATTTTTTATTCGTCG |
| *adeB*-F2 | TGGTTTGACCGTAGTTTC |
| *adeB*-R | TTAAGATGAGATTTTTTTCTTAGAGG |
| *adeB*-R2 | CATAAATGCTGTTGGCCA |
| *adeC*-F | ATGTCTAAATCGACAATCGTATCT |
| *adeC*-R | TTAGACTTTTGATATTCCTCCTCCT |
| *adeR*-F | ATGTTTGATCATTCTTTTTCTT |
| *adeR*-R | TTAGGCGTCATCTTTTACAG |
| *adeR*-R2 | ATAACACTCATGCCTTCACG |
| *adeS*-F | CTGTAAAAGATGACGCCTAA |
| *adeS*-R | AAAATAGCTTTAAAACGATCCAT |
| *adeS*-R2 | GCTTAAAGTCCGTAAGTC |
| *rpsJ-F* | GCAATAGAGTTCGTGGTCAAT |
| *rpsJ-R* | ATACCACATTTGCGACCGAC |
| Primers for identification | |
| *tet(X)*-F[a] | ACGGAAGTTGAAGAAACAGG |
| *tet(X)*-R[a] | ATTCCAAAATGCAATGCACC |
| *tet(X4)*-F | ATGTCGAGCAAGGAAGCAAA |
| *tet(X4)*-R | CATCTCCAAGTAGCGTCACC |
| *tet(X5)*-F | GGGCCAACCCTACACTTC |
| *tet(X5)*-R | TTGGTGATCCACATCTCGT |
| Primers for qRT-PCR | |
| q*adeB*-F | TGGTTTGACCGTAGTTTC |
| q*adeB*-R | CATAAATGCTGTTGGCCA |
| q*adeS*-F | CATGAGTTAAGAACGCCT |
| q*adeS*-R | GCTTAAAGTCCGTAAGTC |

**Supplementary Fig. S1.** The relative mRNA expression levels of *adeB* and *adeS* in the three wild-type isolates (FA56, FA154, and F-1757) and their tigecycline-resistant subpopulations (FA#-R) and mutants obtained after serial passages in the antibiotic-free media (FA#-R-P10, FA#-R-P20, and FA#-R-P30). The expression was measured as a relative quantity by qRT-PCR using the reference gene *rpoB*.

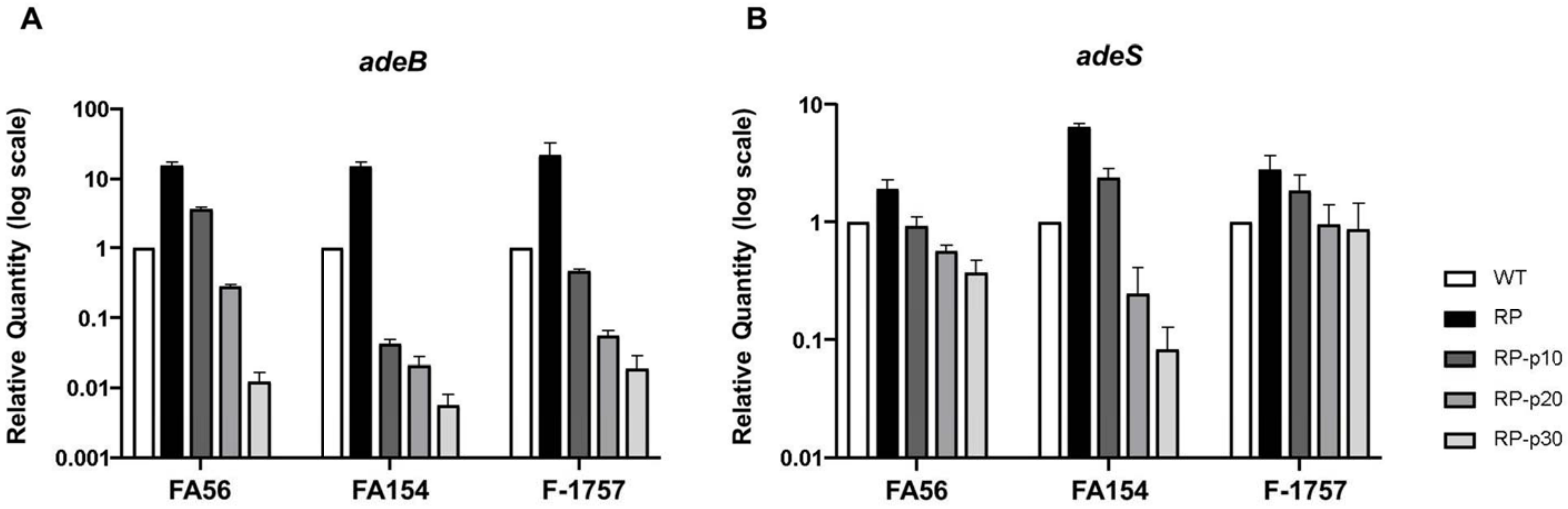

Supplement: SUPPLEMENTAL FILE 1 — Supplemental material. Download SPECTRUM01010-21_Supp_1_seq2.pdf, PDF file, 0.1 MB [file spectrum01010-21_supp_1_seq2.pdf]
